# F117. SCHIZOTYPY PERSONALITY TRAITS RELATED TO PSYCHOLOGICAL FUNCTIONING AND INTERNALIZED STIGMA

**DOI:** 10.1093/schbul/sby017.648

**Published:** 2018-04-01

**Authors:** Olivia Altamirano, Amy Weisman de Mamani

**Affiliations:** University of Miami

## Abstract

**Background:**

People diagnosed with schizophrenia spectrum illnesses report higher levels of internalized stigma in comparison to other mental health diagnoses (Holzinger, Beck, Munk, Weithaas, & Angermeyer, 2003). Studies have shown high overlap between depression and symptoms of schizotypy in nonclinical adolescents (Fonseca-Pedrero, Paino, Lemos-Giráldez, & Muñiz, 2011), but the role that stigma plays in this relationship has yet to be examined. This is of importance because it can be targeted (Rüsch, Angermeyer, & Corrigan, 2005) and prior literature has found that awareness campaigns to reduce stigma can improve psychological functioning (Mittal, Sullivan, Chekuri, Allee, & Corrigan, 2012). Based on previous literature, we predict that schizotypal personality traits will be related to symptoms of depression, anxiety and stress (DASS), and that these will both be related to internalized stigma.

**Methods:**

The current study is a sample of 494 college students who completed surveys to assess for schizotypal personality traits (SPQ; Raine, 1991), depression, anxiety and stress (Depression Anxiety Stress Scales DASS; Lovibond & Lovibond, 1995), and internalized stigma (ISMI; Boyd, Otilingam, & DeForge, 2014).

**Results:**

Correlation coefficients indicated that higher endorsement of schizotypal personality was associated with greater DASS scores (r=.645; p<.01) and internalized stigma (r=.406; p<01). A multiple regression was conducted regressing SPQ on ISMI and DASS, F (2,491) = 183.949, p<.01, R2=.428. Controlling for ISMI scores, DASS was predictive of higher schizotypal personality ratings (Beta=.586; p<01). Controlling for DASS, ISMI scores were also predictive of schizotypal personality (Beta=.123; p=.002).

**Discussion:**

As hypothesized, schizotypy, DASS, and internalized stigma were all positively associated. Internalized stigma could lead to symptoms of depression, anxiety and stress as well as schizotypy. It is possible that internalized stigma plays its own unique role in the onset of schyzotypy. This study is limited by the self-report and cross-sectional nature. Longitudinal studies are necessary to further assess causality in these variables. Screening for schizotypal personality traits when patients present for symptoms of depression and anxiety could be useful in early intervention efforts. Moreover, campaigns that target mental illness stigma could aid in improving psychological functioning, and in reducing schizotypal personality traits.

